# Using predicted length of stay to define treatment and model costs in hospitalized adults with serious illness: an evaluation of palliative care

**DOI:** 10.1186/s13561-021-00336-w

**Published:** 2021-09-20

**Authors:** Peter May, Charles Normand, Danielle Noreika, Nevena Skoro, J. Brian Cassel

**Affiliations:** 1 Centre for Health Policy and Management, Trinity College Dublin, 3-4 Foster Place, Dublin, Ireland; 2 The Irish Longitudinal Study on Ageing (TILDA), Trinity College Dublin, Dublin, Ireland; 3 King’s College London, Cicely Saunders Institute of Palliative Care, Policy and Rehabilitation, London, UK; 4 Massey Cancer Center, Virginia Commonwealth University, Richmond, VA USA

**Keywords:** Palliative care, Hospital costs, Length of stay, Health expenditure, Measurement error, Model comparison, Model selection

## Abstract

**Background:**

Economic research on hospital palliative care faces major challenges. Observational studies using routine data encounter difficulties because treatment timing is not under investigator control and unobserved patient complexity is endemic. An individual’s predicted LOS at admission offers potential advantages in this context.

**Methods:**

We conducted a retrospective cohort study on adults admitted to a large cancer center in the United States between 2009 and 2015. We defined a derivation sample to estimate predicted LOS using baseline factors (*N* = 16,425) and an analytic sample for our primary analyses (*N* = 2674) based on diagnosis of a terminal illness and high risk of hospital mortality. We modelled our treatment variable according to the timing of first palliative care interaction as a function of predicted LOS, and we employed predicted LOS as an additional covariate in regression as a proxy for complexity alongside diagnosis and comorbidity index. We evaluated models based on predictive accuracy in and out of sample, on Akaike and Bayesian Information Criteria, and precision of treatment effect estimate.

**Results:**

Our approach using an additional covariate yielded major improvement in model accuracy: R^2^ increased from 0.14 to 0.23, and model performance also improved on predictive accuracy and information criteria. Treatment effect estimates and conclusions were unaffected. Our approach with respect to treatment variable yielded no substantial improvements in model performance, but post hoc analyses show an association between treatment effect estimate and estimated LOS at baseline.

**Conclusion:**

Allocation of scarce palliative care capacity and value-based reimbursement models should take into consideration when and for whom the intervention has the largest impact on treatment choices. An individual’s predicted LOS at baseline is useful in this context for accurately predicting costs, and potentially has further benefits in modelling treatment effects.

**Supplementary Information:**

The online version contains supplementary material available at 10.1186/s13561-021-00336-w.

## Introduction

Improving care for people with serious medical illness is a policy priority in all parts of the world [1, 2]. This population experiences poor outcomes and high costs in health systems ill-designed to meet their needs, and is growing rapidly in number as populations age [3, 4]. Palliative care is the interdisciplinary specialism that aims to improve pain and symptom management, and communication for people with serious medical illness [5]. In the United States, the dominant model is acute palliative care for hospitalized patients [6].

Economic research on palliative and end-of-life care faces major practical and ethical challenges [7]. Randomized controlled trials are relatively unusual and predominantly occur in the United States [8], where studies are not typically sized for economic evaluation [9]. Observational studies encounter difficulties because treatment timing is not under investigator control and unobserved patient complexity is endemic in routinely-collected data. One common strategy in economic analyses of hospital palliative care has been to control for a patient’s length of stay (LOS), which is associated with both intervention timing and complexity [10]. However, LOS is not an independent baseline predictor but itself an outcome, so this strategy creates a textbook endogeneity problem and biased estimates [10].

While the use of quasi-experimental designs to derive causal evidence from observational data is growing slowly [11–13], a large number of analyses by researchers, payers and hospital programs continue to rely on routine data with no capacity to manage unobserved confounding [14]. An individual’s predicted LOS at admission offers potential advantages in this context. It may allow analysts to model treatment in a way that incorporates intervention timing. It is also a potentially useful marker of patient complexity that has not been widely used in this context.

We aim to evaluate how predicted LOS at admission for a cohort of adults hospitalized with serious medical illness and poor prognosis affects an economic model to estimate palliative care association with hospital costs. Specifically, we have two aims:

**A1.** Does modelling treatment as a function of predicted LOS affect the predictive accuracy of the model, or of the estimated treatment effect?

**A2.** Does including predicted LOS as a covariate affect the predictive accuracy of the model, or of the estimated treatment effect?

## Methods

### Context and rationale

#### Literature review

Cost analyses of hospital palliative care face two serious problems using observational data. First, treatment timing is not under investigator control. Patients are seldom admitted to hospital under a palliative care team but instead subsequently referred to that team. Since patients accumulate costs from the point of admission, but the intervention can only impact treatment choices and so costs from the point of referral, defining a treatment group as any patient who had a palliative care encounter irrespective of timing biases results to the null [15]. In other words, treatment timing is a marker of *capacity* to impact outcome: ceteris paribus, earlier palliative care encounters have a larger treatment effect than later encounters [15–18]. It follows that hospital cost studies should evaluate not simply ‘palliative care’ but palliative care that is provided early in the episode. However no clear clinical definition exists to guide this process.

Attempts to incorporate treatment timing into analysis have consequently been very simple. In a standard retrospective cohort study framework, the treatment groups is defined as ‘did the patient have a first palliative care encounter within *d* days of admission?’, where *d* is decided arbitrarily and sensitivity analyses are performed with different values for *d* [19]. This formulation ‘within *d* days’ is chosen specifically so that all patients had the theoretical opportunity to be in the treatment group. If instead we estimate the effect of a palliative care encounter on *specifically* (e.g.) day four of an admission then any comparison group patient with LOS < =3 was never a candidate for the intervention, and the comparison group cannot be defined by LOS since this is not known at baseline.

The comparison group is then defined as those who did not receive palliative care (“usual care”) and optionally analysts can also retain as comparators those who received palliative care after *d* days in hospital. The decision to include or exclude this later palliative care group in the comparison group is also tested in sensitivity analysis but does not appear to impact results in either direction, likely because late interventions are unable to affect meaningfully total cost of hospital admission [16, 19, 20].

The potential for this approach to define the treatment group sub-optimally are obvious. For example, if an evaluation sets *d* = 3, as has been common [19, 21], then the treatment group may contain patients who stayed in hospital for 3 days and first received palliative care shortly before discharge. Conversely, the treatment group may exclude patients who received palliative care on the fourth day of a very lengthy admission during which there was ample opportunity to change treatment choices and so total costs. In principle, it would be better to model treatment according to *capacity* to effect costs, given what is known at baseline. In a hypothetical sample with observed mean LOS = 9, people receiving palliative care within 3 days of admission on average get the intervention in the first third of their episode. LOS is not known with certainty at admission but if we predict with confidence that a patient would have a LOS = 24 days then s/he could be allocated to the treatment group if receiving palliative care within 8 days of admission (and conversely those with predicted LOS < 9 must have *d* < 3 to be allocated to the treatment group).

The second major problem faced by these studies is systematic selection bias. Those who receive palliative care have a higher illness burden and different preferences for high-intensity treatment that routine data do not capture [22]. Observational studies often find higher mortality rates in the palliative care group than the comparison group [22, 23], but this effect is unobserved in trials [8] except in rare cases where palliative care had a positive survival effect [24]. This unobserved mortality risk may bias cost estimates in either direction: proximity to death is often associated with rising costs [25], so a treatment group with high mortality risk may have higher costs than observed covariates explain; but a clear prognosis that patients are entering the end-of-life phase may precipitate a move away from intensive curative treatment towards supportive care [26], lowering costs in a high-mortality-risk treatment group.

One study of hospital palliative care used an instrumental variable approach, but this relied on the random allocation of physicians to patients in the Veterans Affairs system [13]. In routine US hospital practice, patients choose their physicians and no other cost study to 2018 had reported finding a valid instrument [19]. Early studies estimating the effect of palliative care on cost of hospital admission instead attempted to control for this unobserved complexity by including observed LOS as an additional predictor [10]. However, this leads to a textbook endogeneity problem: LOS is not an independent predictor of costs but rather itself an outcome that is known only when all costs have been accumulated [10].

While there are now some examples of difference-in-differences analyses and related approaches [11, 12, 27, 28], many analyses continue to rely on designs in which intervention timing and unobserved complexity must still be controlled for [14]. An individual’s predicted LOS at admission offers potential advantages in this context. With respect to timing, it may allow the modelling of a treatment variable according to *capacity* to impact total costs and not simply time from admission to first engagement. It is also a potentially powerful measure of patient complexity that has not been widely used in this context.

#### Aim

The econometric challenges laid out in the previous section map directly onto difficulties for policymakers seeking to reimburse or incentivize high-value care for people admitted to hospital with advanced medical illness. Historically there has been no clearly defined method of reimbursement for hospital palliative care services in the US [29], and a lack of payments mechanisms is a key barrier to service development [30, 31]. A binary indicator (“did the patient receive palliative care?”) is a poorly calibrated measure if the effect of that care on outcomes is a function of how early in the admission the patient received that care, how complex were the patients needs, and an interaction between these first two factors. Improving identification of good-value interventions is central to informing value-based reimbursement.

In primary analysis we first build a standard model for evaluating palliative care association with hospital costs, using available data and standard econometric performance measures. We then evaluate how modelling treatment variable using predicted LOS affects model performance and estimated treatment effect, and we evaluate how adding predicted LOS as a covariate affects model performance and estimated treatment effect. We could not identify a reliable marker of predicted LOS in external data; Centers for Medicare and Medicaid Services (CMS) calculated predicted LOS according to diagnosis-related group (DRG) but in our analytic sample the patient DRG is determined only at discharge and is therefore an outcome not a known baseline factor [32]. Instead we derived a predicted LOS metric internally using other admissions in our data. We label this VMLOS. Full details of VMLOS calculation are provided in the Additional file 1: Appendix.

We use real data to understand how our different proposed strategies work in practice, and in doing so we hope to inform future efforts in this field. The alternative – Monte Carlos simulation – would be possible only based on various assumptions about the error distributions of outcome, treatment and other predictors, and we considered unfeasible to design these assumptions in a way that is neutral across modelling approaches [33]. As such we are working with the same data constraints and threats of bias as those who evaluate and reimburse hospital palliative care programs. In the context of the maxim “all models are wrong, but some models are useful” this choice tests practical hypotheses that are hopefully useful without being able to quantify wrongness or compare our estimates to ‘true’ effects.

### Setting and study design

#### Setting

We conducted a secondary analysis of routine administrative data on inpatient admissions from the Virginia Commonwealth University (VCU) Health System. Characteristics of the system and its patient population have been reported previously [18, 22]. Data were collected between 2009 and 2015.

#### Analytic sample

This was a retrospective cohort study. Episodes were eligible for inclusion in the analytic sample if (1) it was the subject’s first admission in the data, (2) the subject had a diagnosis of at least one of seven life-limiting conditions (advanced cancer; heart failure; serious respiratory disease; advanced liver failure; advanced kidney failure; Alzheimer’s disease and related dementias; AIDS/HIV), and (3) the subject had a Van Walraven/Elixhauser index of 20 or higher at admission. The Van Walraven/Elixhauser index is a risk score to predict in-hospital mortality during the admission based on diagnosis of 31 chronic conditions [34]; prior research and assessment of our derivation sample suggests that cohort from this sampling frame with a score of 20+ has an in-hospital mortality rate of approximately 25% and a mean survival from admission of approximately 6 months [21, 34].

Therefore the final sample contained unique individuals with a diagnosis of terminal illness and poor prognosis. We specifically included only those who met the criteria at first admission so that all patients were unique (removing the risk of a small number of people with multiple readmissions influencing results) and those meeting the criteria in later admissions could be used in the derivation sample (see 2.2.3 below). We excluded from all analyses those admitted for a transplant or trauma event, or any person aged under 18 years.

#### Derivation sample

We created a derivation sample in order to calculate a predicted LOS index using site-specific data. Episodes were eligible for inclusion in the derivation sample if (1) the subject was not in the analytic sample, (2) the subject had a diagnosis of at least one of the same seven life-limiting conditions, and (3) the subject had a Van Walraven/Elixhauser score of 13 or higher at admission. Prior research and assessment of our derivation sample suggests that cohort from this sampling frame with a score of 13+ has an in-hospital mortality rate of approximately 10% and a mean survival from admission of approximately 12 months [21, 34].

Therefore the derivation sample contained non-unique individuals with a diagnosis of terminal illness and relatively poor prognosis. We relaxed the requirement for unique patients since we used this sample only for prediction and not treatment effect estimation; while it is possible that a small number of repeat admissions undermine the predictive accuracy of models in the derivation sample, preliminary analyses found this a worthwhile trade-off for the increased sample size. We eased the van Walraven criterion on the same rationale: it increased sample size, which increased predictive power and this offset any loss of predictive power from the derivation sample being on average a little less sick than the analytic sample.

#### Palliative care intervention

The intervention was palliative care from an interdisciplinary team including a physician, advance practice registered nurse, social work, chaplain, and palliative care fellows [35]. Detailed discussion of the roles and purpose of PCU and PCC models have been provided elsewhere [18, 36]. Briefly, palliative care teams provide expert pain and symptom management as well as engaging patients and their families in goals of care discussions and discharge planning. Palliative care engagements can occur in a dedicated unit (PCU) where treatment decisions are under palliative care, and as consultations (PCC) on general wards, where patients remain under the care of other specialisms (e.g. oncology, cardiology) and the PCC teams provides advice. Some patients receive palliative care from both PCU and PCC at different points in an admission. While some differences in treatment effect have been reported, the interventions are substantively similar and provided by the same team within the hospital. For this paper, whose purpose is primarily methodological, we group all PCU and PCC engagements as palliative care and take the first interaction with either to be the first PC engagement for any admission.

### Variables and sources

#### Dependent variable

The dependent variable was total direct cost of hospital admission in 2015 US$. Direct costs are those attributable to a specific patient: supplies, imaging, tests, medications and proportional staffing and equipment costs [37]. Excluded are indirect costs non-specific to individual patients or hospital units, including overall management, security and cleaning. Costs were in US$ and standardized to 2015, final year of data collection, using the Consumer Price Index [38].

#### Primary independent (treatment) variable

The primary independent variable was in all cases a binary variable: did the subject receive early palliative care following admission? Per §2.1.1 there is no independent clinical guidance on defining ‘earliness’. While in principle modelling the treatment as a continuous dose-type effect has advantages, in practice effect estimates tend to be sensitive to underlying assumptions. The distribution of hospital palliative care timing is typically non-normal [16] and the capacity for an intervention to change treatment patterns is not evenly distributed across an admission but instead greatest close to admission, when costs are accumulated disproportionately and treatment decisions are made [39].

To pursue aim A1, we defined for each individual, *i*, two different treatment variables, *t*_i_, according to timeliness. In all cases, people who did not receive palliative care in their admission are in the comparison group (*t*_i_ *= 0*).

Membership of the treatment group depends on when first palliative care engagement occurred. First, we specified a treatment variable *t*_i_^1^ in line with prior studies, based solely on the day, *d*_*i*_, of admission that first palliative engagement occurred [19]:

*If d*_*i*_ *< =3* then *t*_i_^1^ *= 1.*

*If 3 < d*_*i*_ then *t*_i_^1^ *= 0.*

Then we specified an alternative treatment variable that combined *d*_*i*_ and VMLOS.

Mean LOS in our derivation sample was 9.0 days. This implies that the *t*_i_^1^ specification allocates to the treatment group those subjects who on average received palliative care within the first 33% of their admission, and all other subjects are allocated to the comparison group.

We therefore created *t*_i_^2^ based on whether individuals received palliative care within the first 33% of their ***expected*** admission length:

*If [(d*_*i*_*/VMLOS*_i_) *< =(1/3)]* then *t*_i_ *= 1.*

*If [(1/3) < (d*_*i*_*/VMLOS*_i_)*]* then *t*_i_ *= 0.*

We therefore retain late palliative care recipients in the comparison group in all cases. Our main motivation here is to ensure that every treatment effect estimate is drawn from the same analytic sample, and therefore results and diagnostics are due to the changes we impose on the models. Multiple prior studies have found their main results robust to inclusion or exclusion of these late-treatment outliers, who are few in number and belong in the comparison group as people who met the baseline eligibility criteria and received the treatment too late for it to impact outcome.

#### Other independent variables

The basic list of independent predictors comprised those available in the routine administrative hospital data and hypothesized to be associated with outcome, or with treatment and outcome: age (years); sex; race (black; white; neither black nor white); insurance status (Medicare [Medicare fee-for-service or Medicare Managed Care], Medicaid/none [Medicaid, Medicaid Managed Care, self-pay, or unable to pay] and other); primary diagnosis (noncancer, solid tumor, hematological tumor); and first-day admission to the intensive care unit (ICU) or surgery (Table 1). To control for predicted mortality at baseline we calculated an additional predictor: each participant’s Charlson Comorbidity Index (CCI) [41]; CCI is a weighted score of 19 pathologic conditions that is widely used as a prognostic indicator in health services research [42]. Variables were retained if *p* < 0.10 in multivariate regressions; to promote model parsimony variables were binarised if this did not increase the Akaike Information Criterion (AIC).

**Table 1:** Summary of three models, differentiated by specification of independent variables

Model	Primary independent (treatment) variable	Other predictors
***(i)***	*t*^*1*^*:* First PC engagement within three days of admission	All *x*_*i*_ in Table 2
***(ii)***	*t*^*2*^*:* Treatment modelled by VMLOS	All *x*_*i*_ in Table 2
***(iii)***	*t*^*1*^*:* First PC engagement within three days of admission	All *x*_*i*_ in Table 2 plus predicted VMLOS

For details of how *t*^*2*^ was modelled by VMLOS, see §2.3.2

To address A2, we used VMLOS_i_ as an additional predictor.

#### Data sources

Eligible admissions, and accompanying individual-level data on subject characteristics, diagnoses, procedures, dates of admission, were accessed through the VCU administrative database. Diagnoses, traumas and transplant procedures were identified through ICD-9 codes attached to the admission. Palliative care engagements were determined through a free-standing database operated by the palliative care program documenting all patient encounters. Direct costs were extracted from the hospital accounting database at the individual level.

### Models

#### Modelling approach

After finalizing the list of predictors but prior to estimating results, we compared linear and nonlinear regression approaches for heteroscedasticity using the Park test, goodness of fit using the Hosmer–Lemeshow test, link using the Pregibon test and predictive accuracy using the Pearson test [43]. We identified the best approach as a generalized linear model with a gamma distribution and a log link. That is, a model with the following linear function of the regressors:

1 $$ {\eta}_i=\alpha +{\beta}_1{\mathrm{x}}_{i 1}+{\beta}_2{\mathrm{x}}_{i 2}+\cdots +{\beta}_k{\mathrm{x}}_{ik} $$

where the expectation of the response variable is transformed with the following link:

2 $$ {\eta}_i=g\left({\mu}_{\mathrm{i}}\right)=\mathit{\ln}\left({\mu}_{\mathrm{i}}\right) $$

and the conditional distribution of the response variable is specified as proportional to the square of the mean:

3 $$ V\left({Y}_i|\ {\eta}_i\right)={\mu_i}^2 $$

In all regressions, we estimated the average treatment effect on the treated (ATET) with a fixed effects model and bootstrapped robust standard errors (1000 replications) [44]. ATET gives the estimated difference in outcome from the treatment compared to not having had the treatment, holding all other factors in the model constant, but calculates this only on those participants in the treatment group. Thus it compares the treatment group’s observed outcomes with their estimated outcomes had they not received the treatment (i.e. *E[Y*_*1i*_ *− Y*_*0i*_*| D*_*i*_ *= 1]*). This is distinct from the average treatment effect (ATE), which calculates the difference between observed and estimated counterfactual values in both treatment and comparison groups (i.e. *E[Y*_*1i*_ *− Y*_*0*_*]*). ATET is often preferred to ATE in medical studies, where assumptions of true equivalence between treatment and comparison groups in observational studies are less reliable than in trials, and where the ultimate parameter of interest is specifically the effect of the intervention [45]. We checked all models for collinearity and overfitting prior to estimating results.

#### Model specification

In our main analyses we used three models, each employing Eqs. 1, 2 and 3 to estimate associations with the same outcome of interest: total direct cost of hospital admission. The models were differentiated by their choice of predictors, *x*_*i*_, summarized in Table 1.

We specified a default model, labelled *(i)*, mirroring prior practice in this field. We specified an alternate model to address A1, following the default model but employing *t*_*i*_^*2*^ as a treatment variable. And we specified an alternate model to address A2, following the default model but employing VMLOS_i_ as an additional predictor.

#### Bias

Patient characteristics are likely associated with both receipt of palliative care and hospital costs. We were unable to identify an instrumental variable in the data, and so we controlled for observed confounding only using propensity scores [46, 47]. For each treatment variable we weighted treatment and comparison group patients using inverse-probability-of-treatment-weights (IPTWs) from the estimated propensity score [48].

#### Sensitivity analyses

The cut-off for defining *d* in *t*_*i*_^*1*^ and so 33% of LOS in *t*_*i*_^*2*^ is arbitrary and we check our main results to alternative definitions. We also check robustness to use of propensity score weights. See Additional file 1: Appendix.

### Evaluation metrics

We evaluated models on two domains: model accuracy and treatment effect estimation. For model accuracy, we calculated AIC and Bayesian Information Criteria (BIC), R^2^, and in-sample and out-of-sample prediction error [43]. Better information retention and goodness of fit, and lower prediction error, we taken to indicate superior model performance but we did not quantify differences in these metrics using a statistical test.

For treatment effect estimation we examined the width of the confidence interval for each model, where narrower confidence interval indicates superior performance, and we tested whether the derived estimates differed significantly from one another using t-tests.

We had intended to evaluate how the different treatment variables and predictors deliver different magnitudes of observed confounding but the propensity score matching is program is sufficiently powerful that any differences are negligible (Additional file 1: Appendix).

We hypothesized that predicted LOS would improve performance in both analyses.

### Software

Propensity scores were calculated in R [49]; all other analyses were performed in Stata (version 15) [50].

## Results

### Samples

In our timeframe there were 66,760 unique inpatient admissions for adults with one of the seven specified life-limiting illnesses. These admissions were accounted for by 31,791 unique patients.

From these 66,760 admissions, the analytic sample comprised 2674 unique patients who met our criteria: a Van Walraven score of 20+ on their first admission. Our derivation sample comprised 16,425 unique admissions that met our criteria: a Van Walraven score of 13+, and patient not in the analytic sample. We excluded from all analyses 45,220 admissions as meeting criteria for neither sample (i.e. diagnosed with a life-limiting illness but a relatively low probability of mortality), and a further 2441 admissions for analytic sample patients on their second, third, fourth, etc. visit.

Baseline data for the sample, by treatment and comparison per *t*^*1*^, are presented in Table 2. In the unweighted data, there were substantial differences between treatment and comparison group on all factors except gender. The treatment group was older, more likely to be white, more likely to have been admitted via the emergency department and less likely to have been admitted to surgery or the ICU within 24 h. The treatment group was much more likely to have primary diagnosis of cancer, and higher Charlson comorbidity score. Following propensity score weighting, observed differences are negligible.

**Table 2:** Baseline characteristics of the analytic sample (*N* = 2674), before and after propensity score weighting on t^1^

	TG (***n*** = 243)	CG (***n*** = 2431)		Absolute Standardized difference	
		Unweighted	Weighted	Unweighted	Weighted
**Age:** over 75 years	490 (20%)	373 (15%)	490 (20%)	13%	< 0.01%
**Gender:** female	970 (40%)	970 (40%)	971 (40%)	< 0.5%	< 0.01%
**Race:** white	1341 (55%)	1123 (46%)	1341 (55%)	18%	< 0.01%
**Surgery:** first day	100 (4%)	374 (15%)	99 (4%)	39%	< 0.01%
**ICU:** first day	450 (19%)	882 (36%)	449 (18%)	41%	< 0.01%
**Admitted:** via ED	1371 (56%)	1180 (49%)	1371 (56%)	16%	< 0.01%
**1ary dx:** Solid tumor	1130 (47%)	480 (20%)	1131 (47%)	59%	< 0.01%
**1ary dx:** haematological cancer	10 (<0.5%)	58 (2%)	10 (<0.5%)	17%	< 0.01%
**Charlson score:** Mean (SD)	7.5 (2.4)	5.3 (2.9)	7.5 (2.9)	84%	< 0.01%

*TG* Treatment group (*t*^*1*^ = 1), *CG* Comparison group (*t*^*1*^ = 0). Absolute standardized difference compares prevalence for binary variables, and mean and standard deviation for continuous variables, without taking into account sample size. It’s a standard measure of propensity score balance where < 10% is taken as a rule of thumb for acceptable balance [40].

### Treatment variables employing predicted LOS

Sample sizes under different treatment variables are presented in Table 3. Under the default approach, *t*^*1*^, there were 243 (9%) subjects allocated to the treatment group, and under *t*^*2*^, there were 244 (9%). Comparison of treatment group membership shows some divergence between defined treatment groups. There were 211 subjects in both treatment groups. There were 33 subjects for whom *t*^*2*^ *= 1 and t*^*1*^ *= 0*, i.e. they received palliative care on the fourth day of admission or later but as part of a long predicted stay where treatment choices could still be affected. Conversely there are 32 subjects for whom *t*^*2*^ *= 0 and t*^*1*^ *= 1*.

**Table 3:** Sample sizes under different specifications of treatment (*N =* 2674)

	TG ***n***	CG ***n***	TG concurrence with ***t***^***1***^
*t*^*1*^	243	2431	–
*t*^*2*^	244	2430	211 (86%)

*TG* Treatment group, *CG* comparison group. For definitions of t, see Table 1

### Additional variables employing predicted LOS

Summary statistics for predicted LOS are presented in Table 4. Using baseline characteristics, three fifths of the sample (*n* = 1547) were identified as likely short-stay patients (predicted LOS = 7), and one fifth medium stay (predicted LOS = 12) and one fifth long stay (mean LOS = 21).

**Table 4:** Summary statistics of predicted LOS in the analytic sample (*N* = 2676)

VMLOS (categorical)	n (%)	Predicted LOS
*Short-stay*	1547 (58%)	7
*Medium-stay*	584 (22%)	12
*Long-stay*	543 (20%)	21

### Main results

The results for A1 are presented in Table 5. Of nine evaluative metrics, the default model, *(i)*, performs better on seven and one (R^2^) is a tie. It estimates a statistically significant cost-saving from palliative care (ATET: -$11,302; 95% CI: − 14,289 to − 8314), and the estimate under model *(ii)* does not differ significantly.

**Table 5:** Main results for Aim 1: model diagnostics and treatment effect estimation for models differentiated by the use of estimated LOS in defining treatment

	Model diagnostics								Treatment effect estimation			
	R^**2**^	RMSE is	MAPE is	RMSE os	MAPE os	MPE os	AIC	BIC	ATET	95%CI	CI width	t-test
***Model (i)***	0.14	**47,261**	**27,501**	**47,459**	**27,614**	49	**60,450**	**60,515**	− 11302**	−14,289 to −8314	**5975**	t = 0.83
***Model (ii)***	0.14	47,352	27,585	47,562	27,700	**−9**	60,482	60,547	− 9140**	−13,260 to − 5020	8240	

**p < 0.01; *p < 0.05. For explanation of models, see Table 1. *RMSE* root-mean-square error, *MAPE* mean absolute percentage error, *MPE* mean percentage error, *is* in-sample, *os* out-of-sample, *AIC* Akaike Information Criterion, *BIC* Bayesian Information Criterion, *ATET* average treatment effect on the treated, *CI* Confidence interval

The results for A2 are presented in Table 6. The alternative model, *(iii)*, performs better on seven out of nine evaluative measures, with large improvements in R^2^ and prediction error. Again the treatment effect estimate and principal conclusion is unaffected.

**Table 6:** Main results for Aim 2: model diagnostics and treatment effect estimation for models differentiated by the use of estimated LOS as a covariate

	Model diagnostics								Treatment effect estimation			
	R^**2**^	RMSE is	MAPE is	RMSE os	MAPE os	MPE os	AIC	BIC	ATET	95%CI	CI width	t-test
***Model (i)***	0.14	47,261	27,501	47,459	27,614	**49**	60,450	60,515	−11302**	−14,289 to − 8314	**5975**	t = 0.42
***Model (iii)***	**0.23**	**44,875**	**25,066**	**45,116**	**25,196**	−321	**60,058**	**60,135**	− 10365**	−13,490 to − 7241	6249	

For legend, see Table 5

### Sensitivity analyses

We checked the robustness of primary results to different values of *d* in defining our treatment variables, and to use of propensity scores. Conclusions are substantively unaffected. See Additional file 1: Appendix.

**Fig. 1:**
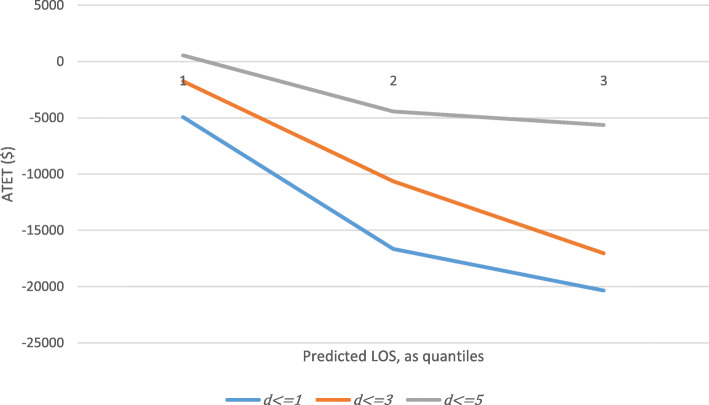
ATET of palliative care, where treatment group membership is defined by different values of d and the sample is defined by tertiles of predicted LOS

In A1, the treatment variable modelled using predicted LOS did not improve model performance but the underlying logic of our hypothesis and motivation remain. As a sanity check, we performed a simple post hoc analysis where the treatment variable was defined according to different values of *d* and the sample was defined according to tertiles of predicted LOS. The results are shown in Fig. 1. For any given sample earlier palliative care (i.e. smaller *d*) is associated with larger cost-savings, per previous studies. For any given definition of treatment, the estimated ATET is larger for those with longer expected LOS. This is in line with our initial hypotheses and suggests residual scope to improve modelling of treatment timing according to expected length of episode as well as time from the start of the episode.

## Discussion

### Key results

This paper aimed to incorporate predicted LOS at baseline in cost analysis of hospitalization for adults with advanced serious illness. We hypothesized that predicted LOS would improve the predictive accuracy and treatment effect estimation of models when employed either in treatment modelling or as a covariate.

For A1, modelling treatment variable as a proportion of expected LOS did not improve model performance or efficiency of treatment effect estimation. This was inconsistent with our initial hypotheses. For A2, including expected LOS as a covariate markedly improved model prediction and efficiency of treatment effect estimation. There were large jumps in information retention, prediction accuracy and goodness of fit. This was consistent with our hypotheses. Predicted LOS is an important predictor of health care use at baseline and investigators using hospital palliative care data should consider seriously using this as a predictor.

### Limitations

The core limitation of this research design is that we are unable to control for unobserved confounding with available data. In particular, while we use CCI as a prognostic variable at baseline and weight the treatment and comparison groups on this variable, proximity to death is hypothesized to be associated with both treatment and outcome but unobserved, and so likely biasing results [23]. However, we do not consider this a major weakness in the context of our aims and methods. Most research studies in this field, as well as much statutory monitoring of hospital performance, are conducted under similar constraints, and our study specifically aims to inform those efforts.

Perhaps a more important limitation is the issue of confounding by timing. It is self-evident that, ceteris paribus, earlier interventions have a larger effect on cost of a hospital admission than later interventions. However in observational data, those who receive palliative care later may differ from those who receive it earlier in unobserved ways. While much consideration has been given to unobserved confounding between those who receive palliative care and those who do not, it is not at all clear how similar are patients who receive earlier versus later palliative care. We have checked our core results to different definitions of treatment by timing to mitigate this concern but it is an important consideration for future work.

Our data are drawn from one hospital site so generalizability of our results are not clear. Previous studies have found broadly consistent results between sites with a palliative care team standardized according to national guidelines, but these are still most common in large urban hospitals and not routine in smaller and more rural settings.

### Interpretation and further research

The primary aim of economic analyses of healthcare interventions is to optimize allocation of scarce resources; to reduce wasteful care and to prioritize good-value care. In palliative care, a strong body of evidence exists that quality of life (QoL) outcomes are at least as good for those who receive the intervention than those who do not [8]. Cost-savings associated with the intervention are therefore interpreted as efficiency gains through changed treatment decisions, on an implicit assumption subject experience in observational cost analyses is equivalent to participant experience in QoL trials [8]. In this context, if we can improve understanding of when and for whom palliative care impacts treatment decisions then this evidence will have important policy implications. For example, specialist palliative care capacity is a scarce resource that ought to be allocated where it is most effective. Reimbursement schemes that aim to reward high-value interventions should incentivize not simply palliative care, but timely palliative care to populations for whom a significant difference is made.

We aimed to supplement a small extant literature on this topic with new approaches to defining treatment variables and estimating the effects of treatment on costs. These efforts were partially successful. Using predicted length of stay as an additional variable on the right-hand side of the model improved noticeably the accuracy of the model, despite being calculated in a relatively small sample using local data. This is a measure that others in the field could consider when estimating and modelling costs within these data constraints. Using predicted length of stay to model treatment variable itself neither changed the estimated results, nor the conclusions, nor the model performance. Our post hoc sensitivity analyses show some encouragement for our prior hypothesis that timeliness should be quantified not simply from admission to interaction, but in the context of predicted LOS, since for any given definition of treatment, the estimated ATET was larger for those with longer predicted LOS.

Future consideration of how palliative care capacity to impact costs varies with length of episode must cross-reference how treatment effect varies by expected LOS with treatment effect variation according to multimorbidity count, given that multimorbidity and LOS are themselves related [19, 51]. Larger sample sizes, wider datasets including biomedical data, and more sophisticated modelling including machine learning would almost certainly improve the predictive accuracy of the LOS index and so the performance of economic models [52].

## Conclusion

Using predicted LOS as an additional covariate in modelling hospital costs for adults with serious illness and poor prognosis improved substantially model performance and the precision of palliative care treatment effect estimates. Using predicted LOS to model treatment variable according to timing of first palliative care interaction did not improve model performance but the scope still exists to yield improvements through this method. Allocation of scarce palliative care capacity and value-based reimbursement models should take into consideration when and for whom the intervention has the largest impact on treatment choices, but further research is required to identify these factors with sufficient precision to inform policy and practice.

## Supplementary Information

**Additional file 1: Appendix Table 1.** Bivariate associations between 31 serious chronic conditions and ln(LOS), derivation sample (*N* = 16,425). **Appendix Table 2.** Bivariate associations between age, gender, diagnosis, admission type and ln(LOS), derivation sample (*N =* 16,425). **Appendix Table 3.** Length of stay in the derivation sample (*N =* 16,425). **Appendix Table 4.** Baseline characteristics of the analytic sample (*N =* 2674), after propensity score weighting for models (ii) and (iii). **Appendix Table 5.** Treatment effect estimates where d = 1, unweighted. **Appendix Table 6.** Treatment effect estimates where d = 3, unweighted.

## Data Availability

Data are not publicly available as mandated by VCU Massey Cancer Center restrictions on healthcare data. Code are not publicly available as there is no public data on which to run code. Queries about code can be directed to the corresponding author.
