# Supplementary material for: Using predicted length of stay to define treatment and model costs in hospitalized adults with serious illness: an evaluation of palliative care
Source: Health Econ Rev. 2021 Sep 20;11:38. doi: 10.1186/s13561-021-00336-w (PMC8454145; doi:10.1186/s13561-021-00336-w)
Supplement: Supplementary file 1 — Additional file 1: Appendix Table 1. Bivariate associations between 31 serious chronic conditions and ln(LOS), derivation sample (N = 16,425). Appendix Table 2. Bivariate associations between age, gender, diagnosis, admission type and ln(LOS), derivation sample (N = 16,425). Appendix Table 3. Length of stay in the derivation sample (N = 16,425). Appendix Table 4. Baseline characteristics of the analytic sample (N = 2674), after propensity score weighting for models (ii) and (iii). Appendix Table 5. Treatment effect estimates where d = 1, unweighted. Appendix Table 6. Treatment effect estimates where d = 3, unweighted. [file 13561_2021_336_MOESM1_ESM.docx]

This Appendix contains three sections, elaborating on methods used in the main manuscript:

1. Derivation of our predicted LOS variable, labelled in the main paper as VMLOS
2. Analytic samples after propensity score balancing, all models
3. Sensitivity analyses: primary analyses ATET

## 1. Derivation of our predicted LOS variable

We followed Van Walraven et al.’s methodology for in-hospital mortality and extended it to LOS.^1^ In the derivation sample (N=16,425), we regressed each of 31 binary variables denoting absence/presence of a chronic disease on log-transformed LOS.^2^ Conditions with a p value greater than 0.05 were discarded. The condition with p<0.05 and the smallest absolute coefficient is given a value of 1 and all other coefficients are scaled accordingly and rounded to the nearest integer (so a condition with a coefficient twice the size of the smallest is given a value of 2, and so on). These values are summed and each individual has an ‘LOS prediction index’. The results of this process are provided in Table 1.

Appendix Table 1 Bivariate associations between 31 serious chronic conditions and ln(LOS), derivation sample (N=16,425)

|  |  | dydx | P value | LOS index points |
| --- | --- | --- | --- | --- |
| 1 | Congestive Heart Failure | 0.21 | <0.005 | 6 |
| 2 | Cardiac Arrhythmia | omitted |  | 0 |
| 3 | Valvular Disease | 0.45 | <0.005 | 13 |
| 4 | Pulmonary Circulation | 0.25 | <0.005 | 7 |
| 5 | Peripheral Vascular Disorders | 0.45 | <0.005 | 13 |
| 6a | Hypertension Uncomplicated | -0.01 | 0.46 | 0 |
| 6b | Hypertension Complicated | 0.08 | <0.005 | 3 |
| 7 | Paralysis | 0.20 | <0.005 | 6 |
| 8 | Other Neurologic Disorders | -0.03 | 0.25 | 0 |
| 9 | Chronic Pulmonary Disease | -0.03 | 0.03 | -1 |
| 10 | Diabetes Uncomplicated | omitted |  | 0 |
| 11 | Diabetes Complicated | omitted |  | 0 |
| 12 | Hypothyroidism | 0.08 | <0.005 | 2 |
| 13 | Renal Failure | 0.09 | <0.005 | 3 |
| 14 | Liver Disease | -0.13 | <0.005 | -4 |
| 15 | Peptic Ulcer Disease | omitted |  |  |
| 16 | AIDS/HIV | 0.20 | <0.005 | 6 |
| 17 | Lymphoma | 0.17 | <0.005 | 5 |
| 18 | Metastatic Cancer | -0.33 | <0.005 | -10 |
| 19 | Solid Tumor without Metastasis | -0.30 | <0.005 | -9 |
| 20 | Rheumatoid Arthritis | 0.17 | <0.005 | 5 |
| 21 | Coagulopathy | 0.53 | <0.005 | 15 |
| 22 | Obesity | omitted |  | 0 |
| 23 | Weight Loss | 0.74 | <0.005 | 22 |
| 24 | Fluid/Electrolyte Disorders | 0.49 | <0.005 | 14 |
| 25 | Blood Loss Anemia | omitted |  | 0 |
| 26 | Deficiency Anemia | 0.27 | <0.005 | 8 |
| 27 | Alcohol Abuse | -0.11 | 0.72 | 0 |
| 28 | Drug Abuse | 0.34 | <0.005 | 10 |
| 29 | Psychoses | 0.17 | <0.005 | 5 |
| 30 | Depression | 0.22 | <0.005 | 7 |

Omitted= zero prevalence in the sample.

We allocated each participant a provisional LOS index score based on Appendix Table 1 and ran a multivariate regression where the outcome was log-transformed LOS, and the predictors were all predictors in the main manuscript plus provisional LOS index score. Outcome data are presented in Appendix Table 2. A collinearity assessment using the –collin- command in Stata established that the mean variance inflation factor (VIF) was 1.18, and the highest individual VIF was 1.48, where mean VIF=6 is a common rule of thumb for concern. We considered risk of collinearity low.

Appendix Table 2 Bivariate associations between age, gender, diagnosis, admission type and ln(LOS), derivation sample (N=16,425)

|  | | dydx | P value | LOS index points |
| --- | --- | --- | --- | --- |
| **Age quartile** | *=2* | 0.03 | 0.07 | 0 |
|  | *=3* | 0.03 | 0.17 | 0 |
|  | *=4* | -0.02 | 0.41 | 0 |
| **Admit type** | *Used neither ED nor ICU* | 0.23 | 0.00 | 2 |
|  | *Used ICU not ED* | 0.33 | 0.00 | 3 |
|  | *Used both ED and ICU* | 0.53 | 0.00 | 5 |
| **Primary dx** | *=solid tumr* | 0.27 | 0.00 | 3 |
|  | *=Heme cancer* | 0.30 | 0.00 | 3 |
| **Van Walr quartile** | *=4* | 0.10 | 0.00 | 1 |
| **Elixhauser quartile** | *=4* | 0.07 | 0.01 | 1 |
| **female** | *=yes* | -0.003 | 0.79 | 0 |

Reference cases for categorical variables: Age quartile=1, Admit type used ED not ICU, Primary dx noncancer

For each participant in the derivation (N=16,425) and analytic samples (N=2,674), we calculated a predicted LOS index score based on the results in Appendix Tables 1 and 2.

We then examined the full distribution of observed LOS in the derivation sample, shown in Appendix Table 3. Mean LOS was 9.0 days, with a median of six days and a long right-hand tail.

Appendix Table 3 Length of stay in the derivation sample (N=16,425)

We categorized the distribution of observed LOS in the derivation sample into tertiles: short stay (<=4 days), medium stay (5-8 days), long stay (9+ days). In the analytic sample we created a new outcome variable according to same cut-points (LOS<=4 | 5<=LOS<=8 | 9<=LOS). In the analytic sample, we regressed the subjects’ predicted LOS index score on this three-level outcome variable, calculating ROC statistic and area under the curve (AUC). Mean AUC = 0.63.

We repeated this process for different categorisations of observed LOS in the derivation sample: 50th and 75th percentiles, 66th and 90th percentiles, etc.. Our best identified AUC = 0.72, cut at 60-20-20. For each subject in the analytic sample, we estimated their LOS according to the observed LOS for derivation sample subjects in the same range of predicted LOS index.

## 2. Analytic samples after propensity score balancing

In the main article we present in <Table 2> the characteristics of the analytic sample before and after propensity score matching for Model (i). Below we present the samples after matching for Models (ii) and (iii). For all three models, differences at the sample level are negligible on observed characteristics.

Appendix Table 4 Baseline characteristics of the analytic sample (N=2,674), after propensity score weighting for models (ii) and (iii)

|  | **Model (ii)** | | | **Model (iii)** | | |
| --- | --- | --- | --- | --- | --- | --- |
|  | **CG (***t^2^*=0**)** | **TG (***t^2^*=1**)** | **ASD** | **CG (***t^1^*=0**)** | **TG (***t^1^*=1**)** | **ASD** |
| **Age:** over 75 years | 22% | 22% | <0.01% | 20% | 20% | <0.01% |
| **Gender:** female | 40% | 40% | <0.01% | 40% | 40% | <0.01% |
| **Race:** white | 55% | 55% | <0.01% | 55% | 55% | <0.01% |
| **Surgery:** first day | 6% | 6% | <0.01% | 4% | 4% | <0.01% |
| **ICU:** first day | 23% | 23% | <0.01% | 18% | 19% | <0.01% |
| **Admitted:** via ED | 52% | 52% | <0.01% | 56% | 56% | <0.01% |
| **1ary dx:** Solid tumor | 38% | 38% | <0.01% | 46% | 47% | <0.01% |
| **1ary dx:** haematological cancer | 0% | 0% | <0.01% | 0% | 0% | <0.01% |
| **Charlson score:** Mean (SD) | 7.0 (3.0) | 7.0 (2.7) | <0.01% | 7.5 (2.9) | 7.5 (2.4) | <0.01% |
| **Predicted LOS:** Medium |  |  |  | 22% | 22% | <0.01% |
| **Predicted LOS:** Long |  |  |  | 20% | 20% | <0.01% |

TG: Treatment group, CG: Comparison group. ASD: absolute standardized difference, compares prevalence for binary variables, and mean and standard deviation for continuous variables, without taking into account sample size. It’s a standard measure of propensity score balance where <10% is taken as a rule of thumb for acceptable balance.^3^

## 3. Sensitivity analyses

We checked the robustness of our results <Tables 5, 6 in the main paper> to different values of *d* in defining our treatment variables, and to use of propensity scores.

Results where *d* =1 are presented in Appendix Table 5. Per our main results, Model (iii) performs best then Model (i) then Model (ii). Conclusions are unaffected.

Appendix Table 5 Treatment effect estimates where d=1, unweighted

|  | **ATET** | **95%CI** | **CI width** |
| --- | --- | --- | --- |
| Model (i), where d=1 | -24405 | -32108 to -16701 | 15407 |
| Model (ii) ,where d=1 | -19705 | -28471 to -10940 | 17531 |
| Model (iii), where d=1 | -19837 | -27072 to -12602 | 14470 |

Results where *d* =3 without propensity scores are presented in Appendix Table 5. Per our main results, Model (iii) performs best. Model (i) and Model (ii) have similar performance. Conclusions are unaffected.

Appendix Table 6 Treatment effect estimates where d=3, unweighted

|  | **ATET** | **95%CI** | **CI width** |
| --- | --- | --- | --- |
| Model (i), no pscores | -17010 | -23304 to -10717 | 12587 |
| Model (ii), no pscores | -8666 | -14950 to -2383 | 12567 |
| Model (iii) , no pscores | -15798 | -21723 to -9874 | 11850 |

## References

1. van Walraven C, Austin PC, Jennings A, et al. A modification of the Elixhauser comorbidity measures into a point system for hospital death using administrative data. *Medical care* 2009;47(6):626-33. doi: 10.1097/MLR.0b013e31819432e5 [published Online First: 2009/05/13]

2. Elixhauser A, Steiner C, Harris DR, et al. Comorbidity measures for use with administrative data. *Medical care* 1998;36(1):8-27. [published Online First: 1998/02/07]

3. Austin PC. Balance diagnostics for comparing the distribution of baseline covariates between treatment groups in propensity-score matched samples. *Statistics in medicine* 2009;28(25):3083-107. doi: 10.1002/sim.3697 [published Online First: 2009/09/17]
